# Monitoring of people and workers exposure to the electric, magnetic and electromagnetic fields in an Italian national cancer Institute

**DOI:** 10.1186/1756-9966-27-16

**Published:** 2008-07-03

**Authors:** Anna Maria Di Nallo, Lidia Strigari, Claudia Giliberti, Angelico Bedini, Raffaele Palomba, Marcello Benassi

**Affiliations:** 1 Laboratorio di Fisica Medica e Sistemi Esperti Regina Elena National Cancer Institute, Rome, Italy; 2 Dipartimento Insediamenti Produttivi ed Interazione con l'Ambiente Italian National Institute for Health and Safety at Work, Rome, Italy

## Abstract

**Background:**

The paper reports the electric, magnetic and electromagnetic fields (*emf*) measurements carried out in the *Regina Elena National Cancer Institute (NCI)*. Several devices, used in diagnostics and in medical cures, can represent sources of *emf *for the workers and for the public subjected to the treatments. The aim is to evaluate their exposition, in order to assess the compliance with the law.

**Methods:**

The investigations have been carried out in the departments of: intensive care, physiotherapy, MR presstherapy and in the surgical rooms. The measurements have been performed using broad band probes in the frequency ranges 5 Hz÷30 kHz and 100 kHz-3 GHz.

**Results:**

The variability of the magnetic induction (B(μT)) levels is between 0,05 μT and 80 μT. The statistical distribution shows that most of the measurements are in the range 0,05<B = 0,5 μT and the 89% of the B(μT) levels are within the 3 μT.

**Conclusion:**

The measurement of the *emf *levels in the *NCI *is recommended because of the presence of the oncological patients; their long stay near the equipments and their day-long exposure represent additional risk factors for which a prudent avoidance strategy have to de adopted.

## Background

In the last years, the increase in the number of systems for telecommunications and for the electric energy transport, has produced a huge debate both in the scientific community and among the public on the potential risks on health due to long time exposition to the electric field (*ef*), magnetic field (*mf*) and electromagnetic field (*emf*) in the working and living environments. The effects produced by the interaction of these physical agents with the biological systems depend on frequency, amplitude of the fields and on the time of the exposition. The effects can be classified in *thermal *and *non-thermal *[1]. The former are scientifically well-know and consist in heat production in the biological system; the exposure limits recommended by the national and international legislation protect from this injury. The existence of the *non-thermal *effects represents an open issue; they concern the evidence of biological changes without an effective increase of temperature. The scientific debate is centred on the issue if a long exposition to *ef-mf-emf *levels lower than the limits could cause harmful effects on health. Many national and international research projects are involved in this investigation by *in vivo*, *in vitro *and epidemiological studies. [2-7]. Until now, conclusive results are still not available.

In Italy, the issue of people exposition to the *ef-mf-emf *is regulated by two decrees promulgated in 2003, one for fields produced by sources with frequencies between 100 kHz and 300 GHz [8], the other for power lines at the frequency of 50 Hz [9]. Their distinctive feature is to establish people long term exposure levels to *ef-mf-emf *lower than the limits recommended by the international standards fixed by the *International Commission on non-ionizing radiation protection (ICNIRP) *[10]. For sources not referable to power lines in the frequency range 0 Hz-100 kHz, the whole of the restrictions fixed by the *European Council Recommendation of 12 July 1999 *[11] has to be considered. About the workers, the directive 2004/40/EC [12], recently incorporated into the Italian legislation system with the decree n. 257/2007 [13], has to be applied.

The main aim of this paper is to report the *ef-mf-emf *measurements carried out in the *Regina Elena National Cancer Institute *(*NCI*). Several electric and electronic devices, used in diagnostics and in medical cures, have been investigated, which can represent potential sources of *ef-mf-emf *for the health staff, for the public subjected to the treatments and also for their accompanists. The main peculiarity of this study is then to verify the exposition to these physical agents for a class of particularly vulnerable patients, as the oncological ones, in order to assess the compliance with the law. The long stay of these patients near the equipments and their day-long exposure can represent additional risk factors for which a prudent avoidance strategy has to be adopted.

## Methods

The investigations have been carried out in the intensive care division, the physiotherapy department, the surgical rooms, the Magnetic Resonance (MR) department and the press-therapy room. For each division, the investigated equipments are reported in Table 1. The study has been extended also to a wireless equipment for internal communications and to external sources.

**Table 1:** Equipments investigated in each division of the *NCI-*IRE

**Divisions**	**Equipments**	**Manufacturer**
**Physiotherapy division**	Electro-stimulators	Biorem TENS
	Electro-stimulators	Biorem ionophoresis (IP)
	Laser therapy	Leve Laser
	Ultrasound therapy	Radar Biorem
	Cyclette	Technogym
	Pressotherapy	Linfopress Fisioline
**Press-therapy room**	Lymphatic Drainage	Linfopress
	Lymphatic Drainage	Linfopress studio
**Intensive care division**	Workplaces – PC	Philips
	Electric panel	FAAE
	Monitor	Philips
	Ventilator	Dräger
	Pump	Abbott
**Surgical rooms**	Ventilator	Dräger
	Electric scalpel	Martin/Conmed
**MR**	Magnetic Resonance	Philips (0,5 T)
**Other departments**	DECT	Alcatel

Initially, the technical characteristics of each equipment have been collected. Moreover, their operating modalities during diagnostic and therapy sessions, and their localizations in each area have been acquired, in order to detect potential interactions.

The measurements have been performed using a broad band probe (EMR 300 Wandel & Goltermann) providing the total electric field for the frequency range 100 kHz – 3 GHz. According with the Italian decrees [8,9], the length of time of each measurement was six minutes. For the frequencies range 5 Hz÷30 kHz, the EFA-3 Wandel & Goltermann, equipped with an external magnetic probe, has been used. All the measurements have been carried out in the operating clinical conditions.

The check on the external sources has been executed using the narrow band device SRM3000 Narda, equipped with an isotropic probe working in the frequency range 75 MHz–3 GHz.

## Results and discussion

### (a) Physiotherapy department

**Figure 1:**
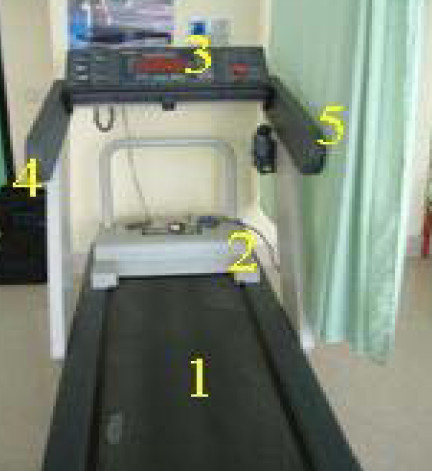
Points of measurements near the *tapis roulant *(detected frequency: 50 Hz).

**Figure 2:**
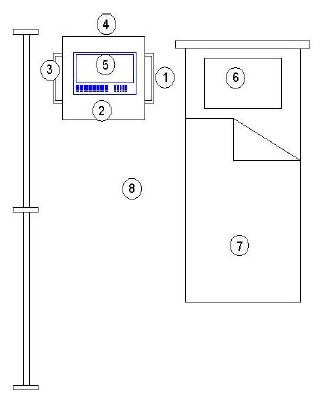
Specific equipments in a physiotherapy box.

The physiotherapy department (*12x3m) *includes an *open space*, where *cyclettes *and *tapis roulant *are positioned, and n. 4 boxes where the devices for specific therapies (Table 1) can be placed on demand. Broad band electric field measurements, carried out in the *open space*, with all the equipments working in operating conditions, have supplied values lower than the sensitivity of the electric field probe (1,0 V/m). In table 2 and 3 are reported the results of the magnetic induction (B(μT)) measurements respectively near the *tapis roulant *(Fig. 1) and specific equipments, conveniently placed in one box (Fig. 2). B(μT) levels are generally lower than 1 μT, with few exceptions, near the *tapis roulant *(4,5 μT), and near the press-therapy equipment, where a maximum B(μT) level of 1,6 μT has been registered. Effective electric field (E_eff_(V/m)) levels lower than 2,3 V/m have been registered near the equipment for ultrasound therapy. Further measurements, performed near the head of the Laser Power Diode, have provided E_eff_(V/m) measurements in the range 1,0 -3,0 V/m.

**Table 2:** The magnetic induction measurements near the *tapis roulant *(fig.1)

**Points of measurements**	**B (μT)**
1	**4,5**
2	0,03
3	0,01
4	0,03
5	0,02

**Table 3:** The magnetic induction B(μT) and effective electric field E_eff_(V/m) levels in the box described in fig. 2

**Points**	**Electro stimulator equipment**		**Press-therapy equipment**	**Ultrasounds therapy equipment**	
				
	**TENS**	**IP**			
	
	**B (μT)**	**B (μT)**	**B (μT)**	**B (μT)**	**E (V/m) (100 kHz÷3 GHz)**
1	0,2	0,2	0,5	0,1	<1
2	0,1	0,1	0,5	0,2	2,3
3	0,07	0,1	1,1	0,1	3
4	0,1	0,1	0,5	0,3	< 1
5	0,3	1,0	**1,6**	0,4	2,2
6	0,02	0,03	0,3	0,5	< 1
7	0,02	0,02	0,03	0,4	< 1
8	0,03	0,02	0,07	0,3	< 1

### (b) Press-therapy room

**Figure 3:**
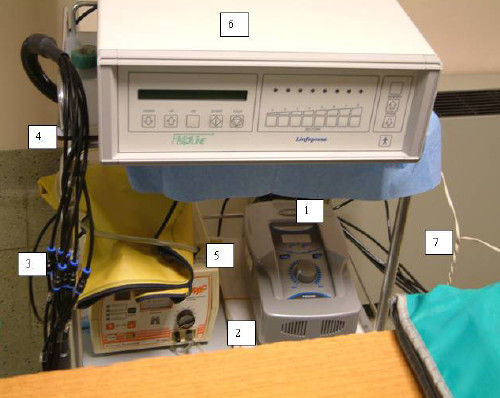
Results of the magnetic induction measurements near the press-therapy equipments.

The rehabilitation area consists of: the reception, the press-therapy room and a medical office. In particular, in the press-therapy room, two equipments for the arms *Lymphatic Drainage *are placed. The duration of this therapeutic treatment varies from 2 hours up to 4 hours and is generally performed for four patients at the same time. The B(μT) levels, measured in the points reported in Fig. 3, are reported in Table 4.

**Table 4:** The B(μT) levels measured in the points reported in Fig. 3

**Points of measurements**	**B (μT)**
1	**80,0**
2	5,3
3	2,0
4	4,8
5	**11,1**
6	2,3
7	1,1

During the treatments, the B(μT) levels detected near the patients are lower than 1 μT. Around the equipments, in positions where only the staff could eventually stay, B(μT) levels between 1,1 and 5,0 μT have been registered. Higher levels (up to 80 μT) have been obtained near the air compressors and the electronics of the equipments, in the positions reported in Table 4. Additional measurements, performed at a height of about 10 cm from the floor, have provided B(μT) levels lower than 1,4 μT. Further investigations, carried out in the contiguous rooms, during the equipments standard working conditions, provided B(μT) values between 0,1 and 0,2 μT.

### (c) Intensive care division

**Figure 4:**
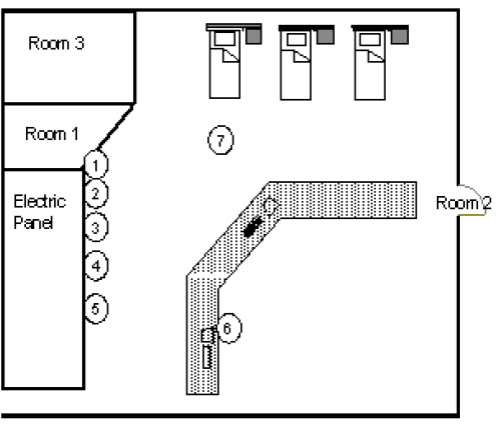
Results of the magnetic induction measurements B(μT) in the intensive care division.

**Figure 5:**
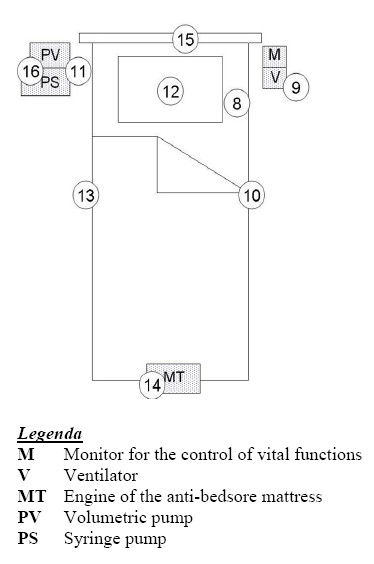
Measurements near a patient's bed in the intensive care division.

The intensive care division consists of an *open space*, where three patients' beds and two monitoring workplaces are located, and three rooms. In Table 5, the results of the B(μT) measurements in the *open space *(Fig. 4) near the sources listed in Table 1, are reported; the magnetic induction measurements, carried out at a height of 1,0 m and 1,9 m from the floor, near the electric panel and near the intensive care staff workplaces, are lower than 1,9 μT. Magnetic field background measurements in the *open space *provided B(μT) levels equal to 70 nT. The high frequencies E_eff_(V/m) values, measured near a patient's bed, resulted lower than 1,0 V/m. In Table 5, the results of the B(μT) measurements around a bed (Fig. 5) are reported. The B(μT) levels close to the patients are lower than 1 μT, while levels up to 7,0 μT have been detected at *1 m *from the bed, at a distance of few centimetres from the volumetric and syringe infusion pumps. Higher B(μT) levels (equal to 14 μT) have been measured at the bottom of the bed near the engine of the anti-bedsore mattress.

**Table 5:** The B(μT) measurements in the open space reported in Fig. 4

**Points of measurements**	**B (μT)**	
	
	H 1 m	H 1.9 m
1	1,7	1,2
2	1,9	1,1
3	0,9	0,7
4	0,3	0,2
5	0,3	0,1
6	0,1	0,1
7	0,07	0,07

### (d) Surgical room

In the surgical room (*6x12 *m), an operating table and two trolleys equipped with ventilators and electric scalpels are placed. B(μT) measurements, performed with the equipments in working conditions, without patients, provided levels of about 50 nT, comparable with the background. Levels up to 24,0 μT have been measured near the electric power of the electric scalpels, at 20 cm from the floor. Broad band E_eff_(V/m) measurements, with all the equipments in working conditions, resulted close to the sensitivity of the electric field probe (1,0 V/m).

### (e) MR room and other internal sources

High frequencies E_eff_(V/m) measurements near the MR and into the gate room during the sequences activation, showed levels lower than 1,0 V/m.

Further investigations have been performed in other departments where the indoor DECT base stations for wireless communication are placed. These apparatus are generally located in the passageways, on the wall at a height of 2 m from the floor. High frequencies E_eff_(V/m) measurements have been carried out at a height of 1,0 m and 1,9 m from the floor close to the DECT systems. The E_eff_(V/m) levels within 1 m from the equipments, at a height of 1,9 m are comprised between 1,0 and 1,3 V/m.

### (f) External sources

**Figure 6:**
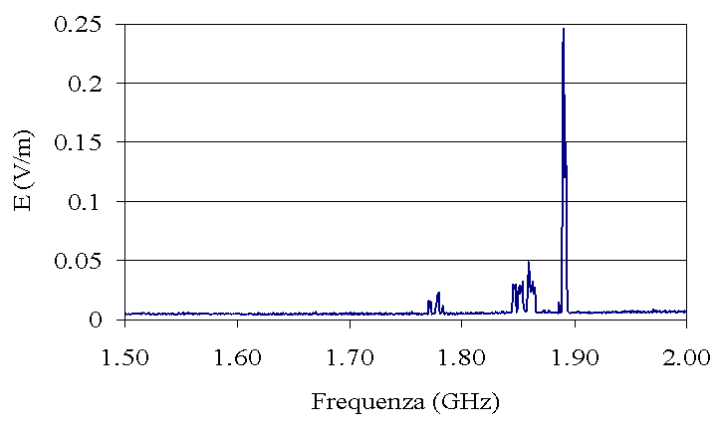
Results of the spectrum analysis carried out in the physiotherapy division.

Further investigations have been carried out outside the *NCI*. In a radius of about 300 m a radiobase station for cellular communications is placed on the roof of a building. In the distance of a few km from the *NCI*, a tower with several broadcasting systems for radio, TV, and cellular radiobase stations is located. Investigations with the broad and the narrow band tools in the external area of the *NCI *show that these sources don't give a significant contribution to the electromagnetic field background in the area. Additional investigations inside the *NCI*, in particular in the physiotherapy division, allowed to detect a weak electric field at the frequency of 1,9 GHz produced by a radiobase station for the cellular networks (Fig. 6).

In Italy, people exposure to the *ef-mf-emf *is defined by two decrees [8,9] in terms of three levels: (i) *limits*, levels that haven't to be exceeded in any circumstances for the protection from short terms effects; (ii) *attention levels *that have not to be exceeded into gambling areas, schools and buildings where people stay for more than 4 hours, as a precaution from the potential risks on health due to long time exposition; (iii) *quality targets*, levels to achieve in a long period in order to minimize people exposure. The respective levels for the protection of people exposure at high and low frequencies are reported in Tables 7 and 8.

The European recommendation [11] establishes *basic restrictions *and *reference levels *for limiting exposure. *Basic restrictions *are restrictions on exposure to time-varying electric, magnetic, and electromagnetic fields which are based directly on established health effects and biological considerations. Depending upon the frequency of the field, the physical quantities used to specify these restrictions are magnetic flux density (B), current density (J), specific energy absorption rate (SAR), and power density (S). The *reference levels *are electric field strength (E), magnetic field strength (H), B, and S. *Reference levels *and *basic restrictions *have been developed following a thorough review of all published scientific literature. The criteria applied in the course of the review were designed to evaluate the credibility of the various reported findings; only established effects were used as a basis for the proposed exposure restrictions. Induction of cancer from long-term electromagnetic fields exposure was not considered. However, since there are safety factors of about 50 between the threshold values for acute effects and the basis restrictions, this recommendation implicitly covers also possible long-term effects in the whole frequency range. The respective levels are reported in Table 9.

The directive 2004/40/EC on the minimum health and safety requirements regarding the exposure of workers to the risks arising from electromagnetic fields, has been issued in the European Official Journal on May 25, 2004, together with its recent application in the Italian regulation [13]. It introduces measures protecting workers from the risks associated with *emf*, owing to their effects on the health and safety. The long-term effects, including possible carcinogenic effects due to exposure to time-varying *ef-mf-emf *for which there is no conclusive scientific evidence establishing a causal relationship, are not addressed. *Exposure limit values *and *action values *have been defined. The former are based directly on established health effects and biological considerations and compliance with these limits ensures that workers exposed to *emf *are protected against all known adverse health effects. The *action values *refer to the magnitude of directly measurable parameters, provided in terms of E, H, B and S; the observance with these values ensure compliance with the relevant exposure limit. *Action values *are obtained from the exposure limit values according to the rationale used by the *International Commission on Non-ionising Radiation Protection (ICNIRP) *in its guidelines on limiting exposure to non-ionising radiation [13].

For the health staff in the *NCI*, the exposure levels are in agreement with the recommendations fixed by the international standards. However, for the health staff, the more restrictive Italian people long term exposure levels have been established as investigation levels. The results of the monitoring in the working areas frequented by patients and accompanies, near the equipments reported in Table 1 show a variability of the B(μT) measurements between 0,01 and 80 μT. The global statistical distribution of the B(μT) levels shows that most of the measurements are in the range 0,05<B = 0,5 μT, with 25% of the levels registered in the surgical room, 9% in the press-therapy room and 15% in the physiotherapy division. The 89% of the B(μT) levels measured near electric and electronic devices used in diagnostics and in medical cures, in positions normally occupied by the patients during the treatments are within 3 μT, while 8% are within 10 μT. Only 3% of the measurements is higher than this level but within the restrictive *limits *imposed by the Italian law to regulate the exposure of the public to the *mf*. The last levels have been measured typically near the engine of the tools and near the electric power of the instrumentations in areas where the presence of the population is not scheduled. In order to minimize or reduce exposures, it is then sufficient to set the area around the devices, with the indications to the staff not to stay close to the devices in their operative conditions.

The exposure conditions of the patients for the extremely low frequencies and the *emf *in the intensive care are in accordance with the measurements carried out by Petrucci [14]. The results show that the type of equipments, their number and also their orientation respect to the patient is important to minimize *mf *levels; in general, a collection of equipments should be avoided and a safety distance have to be adopted to avoid unnecessary exposures.

About the possible electromagnetic interference (EMI) with electronic equipments by radio waves coming from outside the hospital, we find E_eff_(V/m) sensibly lower than the values registered by Hanada et others [15]. However, measurements of the electromagnetic environment should be performed by each hospital specially in urban areas [16] where some sources could potentially induce strong electric field intensity causing malfunctions.

## Conclusion

The monitoring campaign of *ef-mf-emf *levels is suggested in areas equipped with a large number of electric and electronic devices used during the treatments, which can represent potential sources of *ef-mf-emf*. This investigation is also important for the safety of the workers and also for the patients subjected to the treatments and particularly vulnerable, as the oncological patients and also for the accompanists exposures.

The results of the electric and magnetic field measurements, carried out in the *NCI*, near devices normally used in diagnostics and in medical cures, in the departments of intensive care, physiotherapy, MR, press-therapy and in the surgical rooms, show that the exposure levels are lower than that established for the protection of people and workers.

However the monitoring campaign in the hospital environments is recommended when E_eff_(V/m) values beyond the threshold for the electromagnetic compatibility are registered [17-19].

The proper evaluation of the *ef-mf-emf *in the hospital environments allow to map their levels around the equipments, to choose tools with low emission of *ef-mf-emf*, and to design accurately and correctly the working areas, detecting potential interactions, reducing and minimizing the exposures.

## Competing interests

The authors declare that they have no competing interests.

## Authors' contributions

All authors contributed equally to this work, read and approved the final manuscript.

**Table 6:** The B(μT) measurements around the bed reported in Fig. 5

**Points of measurements**	**B (μT)**
8	0,6
9	0,4
10	0,1
11	**7,0**
12	1,0
13	0,1
14	6,0
15	0,7
16	**14,0**

**Table 7:** Limits, levels and quality targets for people exposure to 100 kHz-300 GHz [8]

**Exposure limits**	**Electric field Intensity E (V/m)**	**Magnetic field intensity H (A/m)**	**Power density D (W/m^2^)**
0,1 < f ≤ 3 MHz	60	0,2	-
3 < f ≤ 3000 MHz	20	0,05	1
3 < f ≤ 300 GHz	40	0,01	4
**Attention level**			
0,1 MHz< f ≤ 300 GHz	6	0,016	0,10 (3 MHz-300 GHz)
**Quality targets**			
0,1 MHz< f ≤ 300 GHz	6	0,016	0,10 (3 MHz-300 GHz)

**Table 8:** Limits, levels and quality targets for people exposure to power lines at 50 Hz [9]

	**Exposure limits**	**Attention levels**	**Quality targets**
E_eff _(kV/m)	5	-	-
B_eff _(μT)	100	10	3

**Table 9:** Reference levels for the general public from [11].

**Council Recommendation of 12 July 1999 Reference levels for electric, magnetic and electromagnetic fields (0 Hz to 300 GHz, unperturbed rms values)**				
**Frequency range**	**E-field strength (V/m)**	**H-field strenght (A/m)**	**B-field (μT)**	**Equivalent plane wave power density Seq (W/m^2^)**

0–1 Hz	-	3,2 × 104	4 × 104	-
1–8 Hz	10 000	3,2 × 104/f^2^	4 × 104/f^2^	-
8–25 Hz	10 000	4 000/f	5000/f	-
0,025–0,8 Hz	250/f	4/f	5/f	-
0,8–3 kHz	250/f	5	6,25	-
3–150 kHz	87	5	6,25	-
0,15–1 MHz	87	0,73/f	0,92/f	-
1–10 MHz	87/f^1/2^	0,73/f	0,92/f	-
10–400 MHz	28	0,073	0,092	2
400–2 000 MHz	1,375 f^1/2^	0,0037 f^1/2^	0,0046 f^1/2^	f/200
2–300 GHz	61	0,16	0,20	10
